# Consensus on Criteria for Good Practices in Video Consultation: A Delphi Study

**DOI:** 10.3390/ijerph17155396

**Published:** 2020-07-27

**Authors:** Diana Jiménez-Rodríguez, Diego Ruiz-Salvador, María del Mar Rodríguez Salvador, Mercedes Pérez-Heredia, Francisco José Muñoz Ronda, Oscar Arrogante

**Affiliations:** 1 Department of Nursing, Physiotherapy and Medicine, University of Almeria, 04120 Almeria, Spain; rsd375@ual.es; 2 Emergency Care Unit, Poniente Hospital Health Agency, 04700 El Ejido-Almeria, Spain; 3 Knowledge and Research Management Department, Primary Care District of Almeria, 04007 Almeria, Spain; mariam.rodriguez.salvador.sspa@juntadeandalucia.es (M.d.M.R.S.); rondalia@gmail.com (F.J.M.R.); 4 Research Management Department, Primary Care District Poniente of Almeria, 04700 El Ejido-Almeria, Spain; mmercedesph@yahoo.es; 5 University Centre of Health Sciences San Rafael, San Juan de Dios Foundation, Nebrija University, 28036 Madrid, Spain

**Keywords:** consensus, COVID-19, Delphi method, healthcare providers, telemedicine, video consultation

## Abstract

The use of telemedicine has greatly increased, largely derived from the COVID-19 pandemic, which has created the need for a guide aimed towards the adequate management of a modality of health care: the video consultation. A Delphi study composed of three rounds was conducted with 16 experts in holding video consultations and managing non-technical skills from different specialties and nationalities to conceive a consensus on the criteria needed for properly managing video consultations by healthcare professionals. The consensus criteria were defined by three dimensions (preparation of video consultation, video consultation process, and post-video consultation) and their corresponding items. Excellent consensus data was obtained; therefore, use is recommended by any healthcare professional who is going to utilize a video consultation, in order to manage it effectively.

## 1. Introduction

The pandemic caused by the novel coronavirus disease 2019 (COVID-19) is a threat to global health [1]. We are facing a new disease, and both the health systems and the cultures of the different countries affected, which vary greatly, have had an influence on its management. During the outbreaks of other known infectious diseases, it has been shown that organizational support and the incorporation of new forms of work optimize results in both patients and the level of professional satisfaction [2].

There is no doubt that the COVID-19 pandemic and its rapid evolution has created quite a confounding situation [3], creating great challenges for health systems due to the tremendous pressure that exists to limit the transmission of the virus [4], which has seriously affected all health administrations and policies [5]. In this way, health systems are witnessing, on the one hand, the need to prevent the spread of the virus by minimizing both the exposure of the population and the risk of spread in health centers [1] and, on the other hand, the overload of healthcare services [6]. Among the multiple challenges and changes faced by health systems owing to the COVID-19 pandemic, we find the forceful entry of technology and technological solutions [7], which have promoted the role of telemedicine in healthcare organizations [8]. Consequently, available health care resources will proliferate in the next decade, improving health care services and promoting their implementation in clinical practice and health systems [5]. In this context, telemedicine is presented as a response to the challenge caused by the COVID-19 pandemic, as it avoids overcrowding and human exposure, at the same time that it offers high-quality care [9,10], as indicated in previous studies on public health disasters and emergencies [11].

It should be pointed out that the World Health Organization [12] defines telemedicine as “The delivery of health-care services, where distance is a critical factor, by all health-care professionals using information and communications technologies for the exchange of valid information for the diagnosis, treatment, and prevention of disease and injuries, research and evaluation, and the continuing education of health-care workers to advance the health of individuals and communities". Among the multiple possibilities that telemedicine offers, one is the video consultation modality that consists of an online consultation, where a live interaction is created in real-time through a video call system through which a healthcare professional provides care [13]. Thus, recent studies confirm the increase in video consultations during the pandemic [14] and, consequently, they have gone from being considered as a promising tool [15,16], to currently being a key modality in health care [14,17,18]. In fact, before the pandemic, video consultations were mainly used to provide care for elderly people or those with chronic diseases [19,20], palliative care patients [2,21] and primary care needs [22]. However, the need arose to extend this modality of health care to other clinical conditions and situations during the COVID-19 pandemic, with video consultations now considered to be a perfect solution for numerous patients [18,23].

Video consultations have been used throughout the world [19,24]. Although not all patients can be treated with this modality of health care (owing to a lack of access or inability to use this technology, the severity of the patient’s clinical condition or the need for medical procedures [13]), most patients would benefit from video consultations, since its implementation has shown similar results to face-to-face consultation, improving patients’ health and satisfaction, and improving access to health services [24,25], and video consultations have even resulted in a reduction of costs [24,25,26]. However, it should be noted that creating an effective telemedicine program takes time [18] and various factors must be considered, rather than only taking into account the provision of technological systems. Both healthcare professionals and patients would need technological resources for holding a video consultation (computer, tablet, or smartphone), but they also need to know how to use these. Therefore, healthcare professionals could be trained to act as a ‘tele-facilitator’ [27]. In this sense, it is not only necessary to continuously evaluate and ensure that users acquire the required skills for the use of technologies, but also to provide good practice guidelines to all participants in a video consultation [7].

It is therefore crucial to ensure the quality of health care through this modality. To achieve this, a set of requirements for video consultation assistance is required that deal with factors such as the ethical issues related to the protection of privacy and confidentiality, creating trust in patients, providing competent health care, ensuring continuity of care [13], at the same time without allowing technology to dehumanize the health care provided. Thus, these technologies open new channels of access to health care and offer new opportunities for true patient-centered care where physical distance can be resolved. In this sense, some studies indicate that if the quality of the Internet connection is good, reliable, and stable, patients and healthcare professionals tend to communicate in the same manner as in a face-to-face consultation [28,29]. Accordingly, the next step in video consultations is the study of the skills required to ensure humanized health care in a technological environment. Since respect for the privacy and autonomy of patients, management of emotions, spirituality and effective communication between patients and healthcare professionals [30] are needed for providing high-quality care, emphasizing socio-emotional skills is currently very important and relevant once this modality of health care is established. Consequently, it is necessary to prepare health professionals to understand that video consultations are interactively different from face-to-face consultations in order for them to be prepared to face the main challenges [31], and to thus ensure their adequate adaptation for providing high-quality health care [32].

To date, some approaches have recommended a range of strategies to hold an adequate video consultation, but these are related to quite specific clinical conditions and diseases, and not aimed at providing general health care for most clinical practice situations and patients through this modality. For instance, the following have been promoted: broad guidelines for ethical conduct related to key issues in telehealth/telemedicine [13], an example workflow for inpatient tele-palliative consultation [32], tips on tele-palliative care [28], and finally, recommendations for general use of videos and guides for both patients and healthcare professionals mainly aimed at COVID-19 infection [29]. However, all of these recommendations and guides tend to be quite technical, and no consensus criteria for generalized actions have been reported by experienced healthcare professionals either for video consultations or communication, addressing patient-centered daily clinical practice by video consultation and promoting the non-technical skills required for its adequate management.

Additionally, previous telecare experiences, such as the use of telephone calls, have demonstrated that structured telephone assistance is effective, improving patients’ quality of life and reducing costs [33,34]. Taking all the above into account and considering the current increase of this modality of health care, the main purpose of our study is to elaborate consensus criteria for managing video consultation that contributes to effective and high-quality health care provided by healthcare professionals.

## 2. Materials and Methods 

As the video consultation is a novel and innovative topic, the creation of a consensus on criteria for good practices in video consultation using the Delphi method is proposed, as it is an effective method for obtaining a consensus among experts in any given field of study [35,36]. More specifically, with the use of this method, we intended to reach a consensus among a panel of experts on the guidelines to be utilized to properly manage a video consultation. Consequently, this technique is perfectly adapted to the objective of our study, since it is considered particularly useful for attaining expert consensus on specific subjects, facilitating decision-making, problem-solving or prioritization [37]. In addition, this systematic method is used to create quality indicators or review criteria for areas where evidence alone is insufficient [38] and to synthesize the accumulated opinion of experts. In this sense, it constitutes a group communication between experts through any type of self-administered questionnaire, with no meetings [39], which allows a group of participants who are geographically dispersed to synthesize knowledge [40]. Therefore, the best practice guidelines for the use of the Delphi method proposed by Boulkedid et al. [39] were followed, in relation to the selection of the experts, Delphi questionnaire preparation, survey characteristics, and reporting of results. 

### 2.1. Panel of Experts Composition

According to Mosadeghrad [41], including different stakeholders who often have quite different views on the quality of health care may enrich the results obtained using the Delphi procedure. This author recommends including healthcare professionals, patients or patient representatives and methodologists. In this way, the panel of experts obtained is representative of all stakeholders affected by the results. Consequently, all participants in our study were professionals from different disciplines (psychologists, nurses, and general practitioners) who were national and international experts on the use of video consultations or training in communication skills. In addition, three experts had been patients attended by video consultation. In this way, all experts were selected according to their experience and knowledge of both video consultations and skills required for effective communication, comprising a diverse group of professional and personal profiles. More specifically, only healthcare professionals who had performed at least 20 video consultations were selected to be part of the panel of experts.

### 2.2. Delphi Questionnaires

All questionnaires were sent electronically using the application Google Forms™ (Alphabet, Mountain View, CA, USA), whose use has increased over the years, demonstrating its effectiveness [42]. Moreover, all experts were questioned using an e-questionnaire due to social distancing restrictions during the COVID-19 pandemic, which did not allow in-person interviews. 

### 2.3. Delphi Rounds

Three Delphi rounds were carried out during the study between 25 March and 9 June 2020. 

**First round:** The Delphi process traditionally starts with an open-ended questionnaire to collect specific information about a content area from experts [43]. Consequently, a self-administered questionnaire was electronically sent to participants to obtain their opinion about items that should be included in a guide on video consultations, asking six closed-ended questions about their sociodemographic characteristics and eight open-ended questions related to three themes (Table 1): (1) information before commencing a video consultation, (2) development and closing of a video consultation, and (3) after completion of a video consultation and before starting any other activity. A total of 20 experts were invited, and 16 of them answered the first questionnaire, so the response rate was 80%. In the following rounds, there was a responsible commitment from all the experts who accepted participation in the study, as they completed all the rounds planned (all experts who accepted participation in the first round also participated in the second and third round). Once all the data were obtained, a content analysis was carried out, selecting the elements provided by each expert, its similarities, and frequencies (see the Data Analysis subheading). Subsequently, a proposal for a video consultation guideline was created, using all the information collected.

**Second round:** A second self-administered questionnaire was electronically sent to participants to obtain their opinion about the items that should be included in the guideline, including a brief report of the panel’s most outstanding qualitative results. The response rate was 100% (n = 16). The experts scored this using a Likert scale ranging from 1 (totally disagree with the inclusion) to 4 (totally agree with the inclusion) to assess the inclusion or not of the different items prepared, after analyzing the content of responses obtained in the first-round survey (see the Data Analysis subheading). In addition, an observations section was included, where experts could add indicators or proposals for change or inclusion of new items that they considered necessary to be evaluated in subsequent rounds.

**Third round:** After the second-round survey, both quantitative and qualitative responses and observations were analyzed. The revised survey obtained was forwarded to experts with the same evaluation criteria (Likert scale response of four points to questions and an observations section), including feedback of both quantitative (median, minimal, and maximal ratings) and qualitative (more frequent observations) results obtained in the previous round [39]. Once again, the response rate was 100% (n = 16). Since consensus was achieved, no more rounds were needed.

### 2.4. Data Analysis

A content analysis of data obtained in the first round was performed by two different members of the research team who were experts in qualitative methodologies, by analyzing the eight open-ended questions raised in this round through a self-administered questionnaire. This content was performed according to the methodology proposed by Piñuel [44], which recommends the elaboration, recording and analyzing of all the data obtained from the participants’ discourse, considering the interpretation of those who generated and communicated it. This methodology allowed us to discover the views of each expert by analyzing their responses, selecting analysis units, coding them, and identifying registration units that were translated into the items of the questionnaire used in the second round.

Conversely, both quantitative and qualitative data were obtained in the second and third rounds. Regarding the quantitative data collected, as a 4-point Likert response scale was used, the criteria used to decide the consensus on the inclusion of each element proposed were as follows [35,43]:

- High consensus: the median response was equal to or greater than 3 and at least 80% of the experts scored the element proposed with at least 3 points.
- Low consensus: the median response was equal to or greater than 3 and 70–79% of the experts scored the element proposed with at least 3 points.
- No consensus: the median response was less than 3 and less than 70% of the experts scored the element proposed with at least 3 points.

The major statistics used in Delphi studies are measures of central tendency and level of dispersion in order to present information concerning the collective judgments of the respondents. Generally, the use of median and mode is favored [35,43]. When the median is used, the inclusion of minimal and maximal ratings obtained in experts’ responses to each element is recommended [35,43]. Consequently, the median and minimal and maximal ratings were used in this study as the parameters.

The qualitative data collected in the observations section of the questionnaire distributed during the second and third rounds were also analyzed according to the content analysis methodology proposed by Piñuel [44]. More specifically, in the second round, each basic component of recommendation was evaluated for the purposes of generating the corresponding modification of the item. In the third round, only one recommendation was performed (due to an error in the numbering of an item), so data analysis was not needed. 

### 2.5. Ethical Considerations

Participation was voluntary and the process was anonymous and was carried out following the ethical principles for medical research of the international Declaration of Helsinki [45]. This study is the first phase of a larger project to implement innovative methodologies for training healthcare professionals, which received approval from the Research and Ethics Board of the Department of Nursing, Physiotherapy, and Medicine at the University (EFM No. 75/2020). 

## 3. Results

A total of 16 national (Catalonian (two), Madrid (two), Murcia (three), Galicia (two), and Andalusia (three)) and international experts (Mexico (two) and Argentina (two)) on video consultations participated in the study. The mean age of the participants was 44.31 years (SD = 7.854) and most were women (n = 11; 68.75%). The panel of experts was composed of five psychologists, eight nurses, and three general practitioners (three of these had been in turn patients attended by video consultation). Their average length of work experience was 19.86 years (SD = 9.979), and they had performed 53.43 video consultations on average (SD = 50.029).

After performing the content analysis of the experts’ responses to the eight open-ended questions raised in the first round, responses were translated into the questionnaire distributed in the second round. In this second round, consensus on criteria proposed in this questionnaire was achieved, as more than 80% of the experts scored each element proposed with at least three points, obtaining a median response greater than 3.25. However, to increase the degree of consensus, a third round was carried out after refining several items according to the analysis of both quantitative and qualitative data collected (responses to each item proposed and experts’ observations) in the second round. More specifically, an item was removed (1.3. Verification of what was originally planned) since it was redundant in relation to a previous item, and a total of five items were modified in this round (Table 2 and Table 3):

- Availability of other alternative forms of communication in order to be able to contact the patient in case of problems with the video call (e.g., phone call).
- To plan the reason for the consultation (being flexible, attending to, and prioritizing the needs of the patient), and searching for scientific evidence available to resolve any questions, if necessary.
- To start the conversation, refer to some temporospatial aspect to contextualize (weather, news of the day, etc.).
- Postpone video consultation if it is not possible at that time due to non-rectifiable connection problems that prevent effective communication, but in that case always agree on the date, time and type of the new interaction to provide continuity.
- In the case of performing a prior consultation and if it is necessary, summarize its significant aspects, established objectives, and their degree of compliance, as well as strengths and difficulties found during this consultation before presenting the action plan derived from the current video consultation.

Once again, more than 80% of the experts scored each element proposed with at least three points in this third round, obtaining a median response greater than 3.25. It should be noted that only one expert provided an observation related to an error in the numbering of an item. Consequently, as a high consensus was achieved after this round, no more rounds were needed. 

Therefore, a series of criteria for good practice in video consultation management were obtained by the consensus of a panel of experts. These criteria were defined by three dimensions (preparation of video consultation, proper video consultation process, and post-video consultation). These dimensions and their corresponding criteria are shown in Table 2 (preparation of video consultation), Table 3 (video consultation process) and Table 4 (post-video consultation), including statistical data (median, minimal, and maximal ratings) and the degree of consensus for each item proposed in both the second and third round.

## 4. Discussion

Due to the COVID-19 pandemic, the need has arisen to promote telemedicine, and specifically, video consultation [9,10,14,18]. Consequently, both healthcare professionals and patients must take on these new challenges and changes to adapt to this new situation for continuing to provide high-quality health care and achieving patients’ satisfaction. In this sense, Humphreys et al. [32] indicate that the videoconference interaction with patients and relatives is fundamentally different from in-person interactions. However, according to Calton et al. [28], if the internet connection is adequate, the health professionals and patients tend to communicate in the same manner as in a face-to-face consultation, so effective communication and interaction could be achieved. In addition, other studies state that it is feasible to obtain similar results to traditional face-to-face health care [46,47]. Therefore, it is necessary to train and educate healthcare professionals to eliminate the barriers that may be present, as this is a new modality of health care. In this way, Calton et al. [28] reported that, although the first video consultation implied a major difficulty for healthcare professionals, most of them reported positive experiences in the following video consultations. Aside from mastering the technology required, certain communication and social-emotional skills must be adapted to provide high-quality and humanized health care through this modality. Therefore, we set out to develop consensus criteria on video consultation management, creating a panel of experts to improve health care quality, eliminate technological barriers, and at the same time provide humanized care.

Some specific aspects should be noted in both video consultation phases (preparation of the video consultation, video consultation process and post-video consultation) and the context of the patient-healthcare professional relationship, as it is essential to establish an adequate encounter and interaction between them, generating a substantial impact on the participants’ experience [28]. In this way, consensus criteria developed in our study took the basic elements that influence a video consultation into consideration, such as technical resources, the relationship established between healthcare professionals and patients (verbal and non-verbal communication, attitude, knowledge, and management of difficulties), and the environment during their interaction. In fact, it is precisely because of the environment in a video consultation that the social distance could be reduced (as there are none of the typical barriers found in a healthcare environment such as a desk or an examination table), increasing communication quality and trust between healthcare professionals and patients, and, consequently, promoting the patients’ access to health care while increasing their satisfaction [28]. In this sense, some studies indicate technological difficulties as relevant issues during a video consultation, with the quality and clarity of the audio being a critical point [32], as the understanding of both participants will logically be compromised. In addition, there is a need for adequate lighting and a camera with an effective zoom to facilitate physical examination for an adequate diagnosis and decision-making [48]. Regarding innovations in work environments such as the implementation of a new modality of health care, Humphreys et al., [32] indicate that professionals who are part of healthcare teams have to be kind to each other and that it is necessary to work with humility and patience with both others and oneself. In this study, the consensus of the experts consulted indicated the need to share experiences of the video consultation format with other colleagues, promoting its management by constructive criticism, and without losing patience.

It should be noted that the high consensus obtained in our study was achieved following the recommended methodology criteria for reporting of Delphi studies [35,43]. In addition, the qualitative data collected from experts’ observations were analyzed with a high methodological rigor [44].

Although it may seem controversial to create a standard for a unique and personalized act such as a video consultation, in this study an expert consensus was reached to achieve structured and detailed criteria for the steps and indications that are considered essential in this modality of health care. All of these recommendations are flexible and versatile enough for all healthcare professionals (nurses, general practitioners, psychologists, physiotherapists, nutritionists, etc.) to be able to adapt them to their particular conditions, helping to successfully manage a video consultation. However, it is highly recommended that all healthcare professionals, patients, and caregivers be trained and taught in this modality of healthcare before its real-life use in order to master this technology. In this sense, our guideline includes the availability of other alternative forms of communication in order to be able to contact the patient in case of problems with the video call. In addition, ethical issues related to this technology depend on the specific platform or video consultation software provided by the healthcare service, ensuring privacy and confidentiality. In this case, our guideline includes two elements related to ensuring an environment that is secure, private and confidential. 

Our study is not exempt from limitations, which are fundamentally related to the Spanish and Latin American origin of the experts selected and the sample size (n = 16 experts). Although there is no standard sample size for the Delphi method, some authors recommend that the sample should range between 20–50 experts [49], whereas Delphi’s primary research indicates that the ideal sample is 15 subjects [50]. However, other authors have emphasized that the objective of the study and the available resources are relevant in determining the sample size [38]. In our study, 20 experts were invited, but four did not accept. Consequently, we have prioritized the categorization of ‘expert’ (a person who has mastered the topic of interest, that is, has previous experience with using video consultation), a key requirement in the Delphi technique [37]. In addition, three of the expert participants had a dual expert role, as they had also been a patient in a video consultation, so the wealth of information from experts was evident. Lastly, it would be advisable to include not only other healthcare professionals (such as physiotherapists, nutritionists, etc.) but also patients’ perceptions in future studies to analyze the usefulness of the criteria of good practice in video consultation obtained in our study.

## 5. Conclusions

A consensus on criteria for good practices in video consultations management was achieved with a panel of experts using a 3-round Delphi study. The resulting criteria were focused on both video consultation phases (preparation of the video consultation, video consultation process and post-video consultation) and the context of the patient-healthcare professional relationship. The use of these video consultation criteria is recommended for all health professionals who are going to hold a video consultation. Pilot studies on the use of these criteria for good practice in video consultation management are recommended to analyze their operability, satisfaction, and quality.

## Figures and Tables

**Table 1:** Interview outline.

Aspects Addressed	Questions
**Sociodemographic data**	Age
	Sex
	Country and region
	Professional category
	Work experience (years)
	Approximate number of video consultations
	performed
**Information before a video consultation (before starting it)**	-Indicate the recommendations or previous information that you would give to healthcare professionals who are going to carry out the video consultations before starting them.
	-In the case of patients who are going to be attended through a video consultation, which recommendations or prior information would you give to them before starting it?
**Development and closing of a video consultation**	-Indicate the guidelines that you would give to healthcare professionals to address the beginning of the consultation.
	-Regarding the guidelines to soften the effect of care through a screen and adequately manage the socio-emotional skills of patients, which recommendations would you give to healthcare professionals to provide adequate emotional support, an environment of trust, active listening, show of empathy, and respect, etc.?
	-Indicate the guidelines that you would give to healthcare professionals for adequate management of verbal and non-verbal language during a video consultation
	-In the case of a bad internet connection (e.g., broken audio and/or image), which guidelines would you give to healthcare professionals when technical problems occur?
	-Indicate the guidelines that you would give to healthcare professionals for adequate closing of a video consultation.
**After completing a video consultation and before starting any other activity**	-Indicate the recommendations or advice that you would give to healthcare professionals immediately after completing a video consultation.

**Table 2:** Results of Delphi method: preparation of a video consultation.

Item	Second Round		Third Round	
	Median (Minimal and Maximal Ratings)	Percent of Experts Who Scored at Least 3 Points	Median (Minimal and Maximal Ratings)	Percent of Experts Who Scored at Least 3 Points
1.1.-Resources				
1.1.1.-Sender - receiver:				
-Education and training in communication skills. Verbal and non-verbal language control.	4 (2–4)	93.75%	4 (2–4)	93.75%
-Adapt the message and/or video call to the specific aspects of patient to be attended: personal background and characteristics of the receiver (age, cognitive/sensory/motor deficits, polypharmacy, etc.)	4 (2–4)	93.75%	4 (2–4)	93.75%
1.1.2.-Technical resources				
-Verification of the communication platform: image, sound, internet connection to the network and material or support platforms (such as an electronic medical record or quotation manager) as well as hardware (chargers, headphones, or access to technical support).	4 (2–4)	93.75%	4 (2–4)	93.75%
-Availability of other alternative forms of communication (e.g., phone call). (Item included only in the second round) -Availability of other alternative forms of communication to be able to contact the patient in case of problems with the video call (e.g., phone call). (Item modified in the third round)	4 (2–4)	87.25%	4 (2–4)	93..75%
1.1.3. Environment				
-Prepare the environment: quiet, private, good lighting, no background noise, and free of interruptions.	4 (2–4)	93.75%	4 (3–4)	100%
1.2.-Planning of contents				
1.2.1.-Objectives and contents of video consultation				
-Plan the reason for the consultation to be dealt with by searching scientific evidence available to resolve any questions, if necessary. (Item included only in the second round) -Plan the reason for the consultation to be dealt with (being flexible, attending to, and prioritizing the needs of the patient), searching scientific evidence available to resolve any questions, if necessary. (Item modified in the third round)	3 (2–4)	81.25%	4 (2–4)	87.25%
-Exhaustive review of the patient’s medical history and reason for the consultation, including the results of clinical parameters and/or human responses, establishing realistic expected standards.	4 (2–4)	93.75%	4 (2–4)	93.75%
1.2.2.-Planning registration	4 (1–4)	87.25%	4 (2–4)	87.25%
1.3.-Verification of what was originally planned	4 (1–4)	87.25%	**Item removed** **since it was considered as redundant**	

**Table 3:** Results of Delphi method: video consultation process.

Item	Second Round		Third Round	
	Median (Minimal and Maximal Ratings)	Percentage of Experts Who Scored at Least 3 Points	Median (Minimal and Maximal Ratings)	Percentage of Experts Who Scored at Least 3 Points
2.1.-Resources				
2.1.1.-Sender-receiver				
-Take care of personal appearance, as well as non-verbal language: paraverbal and gestural.	4 (2–4)	93.75%	4 (3–4)	100%
2.1.2.-Technical resources				
-Support available to record information during the video consultation, if possible, without looking up from the screen or image frame.	4 (2–4)	87.25%	4 (2–4)	93.75%
2.1.3.-Environment				
-Generate an environment of safety, avoiding background voices that may undermine confidentiality and privacy.	4 (2–4)	93.75%	4 (2–4)	93.75%
-Environment without distraction	4 (3–4)	100%	4 (3-4)	100%
2.2.-Process				
-Check the connection (correct working of audio and video).	4 (2-4)	93.75%	4 (2–4)	93.75%
-Start with an affectionate greeting, introduction, and mutual identification.	4 (3–4)	100%	4 (3–4)	100%
-Establishment of basic operating rules.	4 (3–4)	100%	4 (3–4)	100%
-Refer to some temporospatial aspect to contextualize (weather, news of the day…). (Item included only in the second round) -To start the conversation, refer to some temporospatial aspect to contextualize (weather, any news of the day…). (Item modified in the third round)	4 (1–4)	81.25%	4 (2–4)	93.75%
-Reduce the technological gap through closeness and complicity.	4 (2–4)	93.75%	4 (2–4)	93.75%
-Approach from superficial to intimate.	4 (2–4)	87.25%	4 (2–4)	93.75%
-Do not assume anything; specify and confirm all inconsistencies or assumptions without speculations.	4 (2–4)	93.75%	4 (2–4)	93.75%
-Active listening. Try not to interrupt or cut the patient’s speech, except to redirect the interview.	4 (3–4)	100%	4 (3–4)	100%
-Continuous feedback.	4 (2–4)	93.75%	4 (2–4)	93.75%
2.2.1.-Contingency management				
-Maintain a proactive attitude to solve problems: look for alternatives and deal naturally with technical problems.	4 (3–4)	100%	4 (3–4)	100%
-Emphasize feedback so as not to miss important details, for example encouraging paraphrasing.	4 (3–4)	100%	4 (3–4)	100%
-Postpone video consultation if it is not possible at that time, but in that case, always agreeing on the date, time, and type of the new interaction to provide continuity. (Item included only in the second round) -Postpone video consultation if it is not possible at that time due to non-rectifiable connection problems that prevent effective communication, but in that case, always agreeing on the date, time and type of the new interaction to provide continuity. (Item modified in the third round)	4 (2–4)	93.75%	4 (3–4)	100%
2.3.-Summary of the most significant aspects and farewell.				
-In the case of performing a prior consultation, summarize its significant aspects, established objectives, and their degree of compliance, as well as strengths and difficulties that occurred during this consultation before presenting the action plan derived from the current video consultation. (Item included only in the second round) In the case of performing a prior consultation, and if it is necessary, summarize its significant aspects, established objectives, and their degree of compliance, as well as strengths and difficulties that occurred during this consultation before presenting the action plan derived from the current video consultation. (Item modified in the third round)	4 (1–4)	93.75%	4 (3–4)	100%
-Summarize all of what has been discussed and remind of planned objectives and agreed upon interventions to achieve them.	4 (2–4)	81.25%	4 (2–4)	93.75%
-Request feedback to verify that the most relevant information has been understood.	4 (2–4)	93.75%	4 (2–4)	93.75%
-Ask the patient if he/she has felt comfortable during the video consultation.	4 (2–4)	93.75%	4 (2–4)	93.75%
-Schedule the next appointment, give thanks for the collaboration and farewell.	4 (3–4)	100%	4 (3–4)	100%

**Table 4:** Results of Delphi method: post-video consultation.

Item	Second Round		Third Round	
	Median (Minimal and Maximal Ratings)	Percentage of Experts Who Scored at Least 3 Points	Median (Minimal and Maximal Ratings)	Percentage of Experts Who Scored at Least 3 Points
3.1.-Process				
3.1.1.-Assessment				
-Evaluation using established tools in the planning (pre-video consultation) for both the communication process and specific objectives of the video consultation.	4 (1–4)	93.75%	4 (3–4)	100%
-Review unforeseen incidents to analyze their solution.	4 (3–4)	100%	4 (3–4)	100%
-Identify aspects of the video consultation that were not satisfactory or that could be done otherwise. Consult other peers and encourage constructive criticism.	4 (2–4)	87.25%	4 (2–4)	93.75%
3.1.2.-Registration				
-Registration of information, administrative procedures (prescriptions, referrals, requests for medical tests, etc.), and management of next consultation.	4 (3–4)	100%	4 (3–4)	100%
-Send the supporting documentation and, if not possible, record it on the agenda so as not to forget.	4 (3–4)	100%	4 (3–4)	100%
3.3.-Resources				
3.3.1.-Sender				
-Take a break (video consultations are more exhausting than face-to-face consultations). Breathe, move, and look up from the screen.	4 (2–4)	93.75%	4 (2–4)	93.75%
3.3.2.-Technical resources				
-Perform an adequate disconnection process	4 (1–4)	93.75%	4 (2–4)	93.75%
3.3.3.-Environment				
-Leave physical space in optimal conditions and clear to be prepared for the next use.	4 (3–4)	100%	4 (3–4)	100%
